# Rumination in a Boy of Nine Years*Read before the Philadelphia Pediatric Society, June 13th, 1899.

**Published:** 1899-09

**Authors:** Luther C. Peter

**Affiliations:** Philadelphia, Pa.


### RUMINATION IN A BOY OF NINE YEARS.*

By LUTHER C. PETER, M. D., PHILADELPHIA, PA.

The case which I bring before you is the subject of that interesting and rare phenomenon of disease, if I may so designate it, rumination or mericismus.

He is a bright boy, nine years of age, of a quick, nervous temperament, the oldest of six children, all of whom are nervous, but more robust than our patient. The father is intemperate, and the mother is subject to attacks of migraine, and seems to have bequeathed to her children an unstable, excitable disposition. Two years ago the parents noticed that the child, after leaving the table, made some slight effort, as in vomiting, and continued to chew and swallow. The habit was also observed by his playfellows, and the boy was jeered by his companions and corrected by his parents, but to no avail—he persisted in the practice. His own account is to the effect that he accidently discovered that he could bring up his food after eating, and since it tasted just as when he swallowed it the first time, he rechewed and swallowed it. Later, however, the food came up apparently without effort on his part, and in fact when he did not desire it. For some minutes after eating, the food regurgitated is sweet, and he can recognize the various articles of diet, but after a time it becomes sour, and he is compelled to reject it. Although constantly corrected by his parents, he persisted in the practice, partly because he could not stop it without much effort and partly because he liked the habit. It is his usual custom to reswallow the food, but reject it when sour, unless he is in school or some place where this is not possible. His mother says he has grown thin, and is so nervous of late that he cannot be corrected for misconduct. His appetite is poor, his diet consisting of little else than meat, of which he is very fond.

The appearance of the food varies much with the amount

---

* Read before the Philadelphia Pediatric Society, June 13th, 1899.

of time that intervenes between the ingestion and the regurgitation. Soon after eating, it consists of finely divided solid particles, not disagreeable in odor; an hour later it is semifluid and of a sour odor. There is nothing abnormal in its appearance, and the digestive process seems to progress naturally. Dr. Kane, of St. Christopher's Hospital, made a number of examinations of the vomited matter, and invariably found hydrochloric acid present one hour after the food was eaten. It is quite evident from these tests that the food passes directly into the stomach after being swallowed; and furthermore, repeated examinations with the bougie, to which the patient submits readily, failed to reveal an obstruction or pouch, which is alleged in some cases to act as a receptacle for the food when first eaten.

When he came to the hospital he could at times, with great difficulty, prevent the regurgitation of food, and could reproduce his breakfast at will when requested. Under a tonic sedative, and suggestive line of treatment, the boy has practically overcome the habit, and now has it under control.

The condition is a rare one, even in adult life, and in children one may judge of its rarity by a total absence of any discussion of the subject in most of our modern text-books on diseases of children. Among children the phenomenon occurs most frequently in idiocy, and in these subjects is usually caused by boulimia. Dr. James Hendrie Lloyd, in discussing a paper read by Dr. David Riessman on this subject (Proceedings of Philadelphia Neurological Society, 1895) cited a number of cases which occurred at Elwyn during his term of service, in some of whom the habit was most disgusting. It does, however, occur in children of normal mental development, in whom, however, as in adults, there is a history of some disturbing nervous element. Sommers reported a case, five years of age (*N. Y. Med. Record, 1897*), associated with hydrocephalus. Singer (*Deutsche Archiv. f. Klin. Med., 1893*), described two cases of sixteen and seventeen years of age, in whom the fields of vision were much contracted, and other nervous phenomena marked. Another case of seven years, reported by Runge (*Boston M. & S. F., 1895*), was the third member of a family who had acquired the habit—his father and grandfather both having been victims of rumination. Holliday (*N. Y. Med. Record, 1897*) gives an interesting account of his own case. He had acquired the habit in early childhood and continued until he was eighteen, when he learned to smoke. Smoking after each meal took away the desire to regurgitate.

The vast majority of cases occur among men, physicians and students being the most frequent subjects. It usually is associated with a profoundly neurasthenic condition or idiocy, and is therefore classed as a neurosis. To this rule our patient is not an exception. He is rather young to be a victim of neurasthenia, but springs from a neurotic ancestry, and is distinctly a nervous child, as his every movement indicates. He is also a great lover of meats, a peculiarity which is shared by most of these patients. His generally poor physical condition is, no doubt, due to his pernicious habit. He was compelled of late by his parents to reject the food when it regurgitated, and, being a light eater, he probably has not received much nourishment.

The therapeutics employed, which were Blaud pill, bromide salts, 15 grains daily, and suggestion have accomplished much good. His general health is improving; he is still nervous, but his habit is broken. It is generally thought that the habit is within the control of the will, and the termination of a majority of the cases warrants this conclusion. Trephining has been advocated and practiced by some to effect cure. The cases benefited are probably not cured by the trephining, but by mental impression, and as Dr. Lloyd expresses it (Proceedings of Philadelphia Neurological Society, 1895): It does not seem to be necessary "to cut a hole into a man's skull in order to put a suggestion into his mind."

CONCLUSIONS.

1. Rumination is a neurosis, associated with a profound neurasthenic condition or idiocy.

2. It is not, as a rule, associated with a diverticulum or dilatation of the lower end of the esophagus, but is primarily a stomach neurosis.

3. It may at times be hereditary.

4. It occurs more frequently in males than in females.

5. It usually is within the control of the will.

6. The prognosis as to cure is good.—*Pediatrics*, July 15th, 1899.

